# Dynamic Assessment of Systemic Inflammatory Markers in Predicting Pathological Complete Response After Neoadjuvant Treatment in Triple-Negative Breast Cancer

**DOI:** 10.3390/jcm15145614

**Published:** 2026-07-17

**Authors:** Grzegorz J. Stępień, Katarzyna Boguszewska-Byczkiewicz, Maria Wołyniak, Monika Ryś-Bednarska, Thomas Wow, Agnieszka Kołacińska-Wow

**Affiliations:** 1 Department of Oncological Physiotherapy, Medical University of Lodz, Paderewskiego 4, 93-509 Lodz, Poland; 2 University Clinical Hospital No. 2 of the Medical University of Lodz, 90-549 Lodz, Poland; 3 Department of Surgical Oncology, Nicolaus Copernicus Provincial Multispecialty Center for Oncology and Traumatology, 93-513 Lodz, Poland; 4 Department of Biostatistics and Translational Medicine, Medical University of Lodz, 92-215 Lodz, Poland; 5 Department of Clinical Oncology, Nicolaus Copernicus Provincial Multispecialty Center for Oncology and Traumatology, 93-513 Lodz, Poland; 6 Medical Practice Thomas Wow, 00-841 Warsaw, Poland

**Keywords:** triple-negative breast cancer, neoadjuvant chemotherapy, Pan-Immune-Inflammation Value, pCR

## Abstract

**Background/Objectives:** Triple-negative breast cancer (TNBC) is an aggressive subtype in which neoadjuvant chemotherapy plays an important role in initial treatment. Pathological complete response (pCR) following neoadjuvant therapy can serve as a surrogate marker for long-term survival. Our study aimed to evaluate the value of the Pan-Immune-Inflammation Value (PIV), Neutrophil-to-Lymphocyte Ratio (NLR), and Platelet-to-Lymphocyte Ratio (PLR), measured at multiple time points, in predicting pCR. **Methods:** We retrospectively included 89 patients with non-metastatic TNBC treated with neoadjuvant chemotherapy with or without immunotherapy, followed by surgery. Neutrophil-to-Lymphocyte Ratio (NLR), Platelet-to-Lymphocyte Ratio (PLR), and Pan-Immune-Inflammation Value (PIV) were calculated at baseline, before the second treatment cycle, and at the pragmatic pre-transition assessment before a subsequent treatment phase, when applicable. The primary endpoint was pCR, defined as ypT0N0. Multivariable logistic regression models included age, Ki-67, clinical T stage, and tumor grade. Biomarker-extended models were compared with the clinical model on identical complete-case samples. Model performance was assessed using the area under the receiver operating characteristic curve (AUC), likelihood ratio testing, calibration measures, Brier scores, and bootstrap internal validation with 2000 resamples. **Results:** pCR was achieved in 25 of 89 patients (28.1%). Baseline platelet counts were lower in patients with pCR than in those without pCR (median 246 × 10^3^/µL vs. 272 × 10^3^/µL; *p* = 0.021). At the pre-transition assessment, patients with pCR had lower monocyte counts (0.20 × 10^3^/µL vs. 0.57 × 10^3^/µL; *p* = 0.048) and lower PIVs (378.9 vs. 746.0; *p* = 0.011). Baseline PIV did not improve the clinical model. On the same 83-patient sample, adding baseline platelet count increased the apparent AUC from 0.751 to 0.807; however, the bootstrap 95% confidence interval (CI) for the AUC difference included zero (−0.003 to 0.128). On the same 75-patient sample, adding pre-transition PIV increased the apparent AUC from 0.735 to 0.798, with a bootstrap 95% confidence interval for the AUC difference of 0.005 to 0.142. The optimism-corrected AUC for the overall clinical model was 0.716, indicating lower internally validated performance than suggested by the apparent AUC. A smaller absolute increase in PIV from baseline to the pre-transition assessment was associated with pCR, but this finding was definition-dependent and remained exploratory. **Conclusions:** Standalone baseline markers have limited utility in predicting pCR in non-metastatic TNBC. Pre-transition PIV showed the most consistent incremental association with pCR beyond conventional clinical variables, whereas the added value of baseline platelet count was uncertain and baseline PIV provided no incremental benefit. Because of the retrospective design, limited sample size, treatment heterogeneity, and evidence of model optimism, these findings should be regarded as hypothesis-generating and require external validation.

## 1. Introduction

According to the latest global reports, breast cancer (BC) is the most prevalent female malignancy, accounting for 23.8% of cancer cases among women [1]. BC constitutes a heterogeneous disease that can be divided into five subtypes based on immunohistochemical status: luminal A, luminal B HER2-positive (human epidermal growth factor receptor 2-positive), luminal B HER2-negative, HER2-enriched, and triple-negative breast cancer (TNBC) [2]. The latter, which represents approximately 15–20% of all invasive breast cancers, is characterized by the lack of estrogen receptor (ER) and progesterone receptor (PR), as well as HER2 expression [2,3,4]. Furthermore, TNBC is an aggressive breast tumor, associated with worse overall survival (OS) and higher recurrence rates, compared to non-TNBC types [2,3,5,6].

Due to its unfavorable biology and lack of specific therapeutic targets, neoadjuvant chemotherapy (NACT) based on anthracyclines followed by taxanes, with the addition of pembrolizumab when indicated, plays a pivotal role in the initial treatment of non-metastatic cT1c-T4 or cN+ TNBC. This treatment modality is intended to downsize cancer and thereby enable tumor resection and, if possible, facilitate the least invasive surgical approach (breast-conserving surgery) [7,8,9]. To evaluate the efficacy of this modality in clinical trials, pathological complete response (pCR) has been introduced as a surrogate marker for long-term clinical outcomes. Achieving pCR in surgical specimens following neoadjuvant chemotherapy has been associated with improved overall survival (OS) and event-free survival (EFS) [10,11,12].

TNBC represents the most immunogenic breast cancer type [3,13]. To date, several tissue-based and genetic biomarkers have been established or are being actively explored. Higher levels of tumor-infiltrating lymphocytes (TILs), reflecting an active anti-tumor immune microenvironment, have been linked to lower recurrence rates after NACT and may predict pCR [14,15]. Programmed death-ligand 1 (PD-L1), expressed on the surface of tumor and immune cells, is a ligand for programmed death-1 (PD-1), expressed on active T cells. The interaction between these proteins plays a crucial role in regulating inflammation and anticancer responses [16,17]. PD-L1 expression status has emerged as a crucial predictive biomarker, as recent advances in immunotherapy have led to the introduction of PD-1/PD-L1 inhibitors that increase cancer-specific responses and the penetration of T cells into the tumor microenvironment [16,17]. Determining this status is important for selecting patients who may benefit from the addition of PD-1/PD-L1 inhibitors, such as pembrolizumab, to conventional chemotherapy regimens in TNBC. This advancement in TNBC therapy significantly increases pCR rates, especially among lymph node-positive patients (N+), and reduces the risk of recurrence [17,18]. Furthermore, BRCA mutation status is not only valuable in genetic counseling but also influences sensitivity to specific DNA-damaging agents, such as platinum-based neoadjuvant regimens. Recent studies have shown that patients with BRCA mutations exhibit higher pCR rates [19,20]. Another approach for predicting pathological complete response in TNBC is the detection and monitoring of circulating tumor DNA (ctDNA). Clearance of ctDNA after four cycles of anthracycline-based NACT was significantly associated with pCR. Conversely, detection of ctDNA at this time point was linked to poorer overall and recurrence-free survival [21].

Nonetheless, assessing such biomarkers requires additional time, increased costs, and specialized laboratories. Therefore, several studies have evaluated easily accessible blood markers, such as Neutrophil-to-Lymphocyte Ratio (NLR), Platelet-to-Lymphocyte Ratio (PLR), or Pan-Immune-Inflammation Value (PIV), for their ability to predict pCR and clinical outcomes in breast cancer [22,23,24,25], as well as other solid tumors treated with neoadjuvant therapy [26,27,28]. However, these markers are usually measured only once, at treatment initiation, and to the best of our knowledge, their predictive value remains insufficiently explored and only a few studies have specifically evaluated their potential in TNBC. Furthermore, the existing literature frequently analyzes these laboratory parameters in isolation, rather than quantifying their predictive value when integrated into comprehensive, multivariable clinical models.

The aim of this retrospective study was to assess the role of NLR, PLR, and PIV measured at different treatment stages in predicting pCR in women with non-metastatic TNBC undergoing neoadjuvant treatment. Additionally, we assessed whether these markers added predictive value to conventional clinicopathological variables.

## 2. Material and Methods

### 2.1. Study Population

We retrospectively identified patients diagnosed with TNBC who underwent mastectomy or breast-conserving surgery (BCS) with sentinel lymph node biopsy (SLNB) or axillary lymph node dissection (ALND) between October 2017 and September 2024 at the Breast Cancer Unit of the Nicolaus Copernicus Provincial Multispecialty Center for Oncology and Traumatology in Lodz, Poland. Medical records were reviewed, and the clinicopathological data, including age, comorbidities, medical history, pathological characteristics (PR, ER, HER2, Ki-67, tumor grade, histologic type, presence of multifocality or DCIS, and pathological TNM stage), pretreatment clinical TNM (cTNM) stage assessed according to the current guidelines, details of administered chemotherapy and immunotherapy, as well as laboratory data were retrieved and analyzed. The clinical stages were assessed based on the TNM Classification of Malignant Tumors, UICC 8th edition.

The inclusion criteria were: (1) histopathologically confirmed TNBC by core needle biopsy; (2) receiving neoadjuvant chemotherapy ± immunotherapy followed by surgical treatment.

The exclusion criteria were: (1) male breast cancer; (2) metastatic disease (stage IV); (3) bilateral or synchronous breast cancer; (4) breast cancer diagnosed during pregnancy; (5) previous breast cancer diagnosis.

Following the initial diagnosis, each patient was individually evaluated during an interdisciplinary consultation at our institution and was provided with an up-to-date neoadjuvant treatment plan (chemotherapy ± immunotherapy) tailored to their specific case and medical condition, in accordance with the standard of care. Neoadjuvant treatment was administered in different combinations, according to multidisciplinary team recommendations and the treating physician’s judgment. Predominately, the treatment protocol consisted of chemotherapy—AC (doxorubicin with cyclophosphamide) + taxane (paclitaxel or docetaxel), or chemoimmunotherapy—pembrolizumab + taxane + carboplatin followed by pembrolizumab + AC.

After neoadjuvant treatment, patients could undergo either mastectomy or breast-conserving surgery, with axillary lymph node dissection or sentinel node biopsy, as well as additional therapy, e.g., radiotherapy, not evaluated in our study.

For the primary tumor (T status), regression was defined as a decrease from the clinical to the pathological stage (cT > ypT), stability as no change (cT = ypT), and progression as an increase (cT < ypT). Accordingly, regression in nodal status (N status) was defined as a decrease from the clinical to the pathological stage (cN > ypN), stability as no change (cN = ypN), and progression as an increase (cN < ypN). At the overall disease level (disease stage), regression was defined as a decrease in the overall prognostic stage (clinical stage > pathological stage), stability as no change, and progression as an increase or the development of distant metastases.

The Ethics Committee of the Medical University of Lodz, Poland, stated that our research does not need to be evaluated by the Ethics Committee, because of its retrospective nature (RNN/180/23/KE; 11 July 2023).

### 2.2. Histopathological Assessment

Biopsy samples were routinely evaluated by pathologists. ER and PR status were considered negative if less than 1% of tumor cell nuclei showed staining. HER2 status was negative if the immunohistochemistry (IHC) score was 0 or 1+, or if the IHC score was 2+ without evidence of *ERBB2* gene amplification by in situ hybridization. Ki-67 index was also analyzed, with the percentage of positively stained tumor cell nuclei used as an indicator of proliferation.

Pathological complete response (pCR) was defined as the absence of both invasive cancer and cancer in situ in the breast and axillary lymph nodes (ypT0N0). An extended definition of pCR, permitting the presence of cancer in situ in the breast (ypT0/isN0), was also analyzed.

### 2.3. Inflammatory Markers Analysis

Peripheral blood samples were collected from our institutional database and analyzed at three time points: before the initiation of neoadjuvant treatment (before the first cycle), after the first cycle (before the second cycle), and at the pre-transition assessment, when applicable. The pre-transition assessment was defined as the last complete blood count available before the transition to the subsequent neoadjuvant treatment phase. Because treatment sequencing differed between regimens, this time point did not necessarily correspond to the same chemotherapy cycle in all patients and was therefore analyzed as the pragmatic pre-transition marker. Laboratory values were clinically used as part of patient qualification for the respective treatment cycle.

All leukocyte subsets and platelet counts were converted to 10^3^/µL before calculation of inflammatory markers to ensure consistent scaling. Inflammatory markers were calculated using complete blood count parameters according to the following formulas: Neutrophil-to-Lymphocyte Ratio (NLR): NLR = neutrophils/lymphocytes, Platelet-to-Lymphocyte Ratio (PLR): PLR = platelets/lymphocytes, Pan-Immune-Inflammation Value (PIV): PIV = (neutrophils × platelets × monocytes)/lymphocytes. Because all components were expressed in 10^3^/µL, PIV is reported as an arbitrary unitless value.

### 2.4. Statistical Analysis

Continuous variables were summarized using the median and interquartile range or range, as appropriate, and categorical variables were presented as counts and percentages. The distribution of continuous variables was assessed using the Shapiro–Wilk test. Because most laboratory variables were non-normally distributed and the sample size was limited, comparisons between patients who achieved pathological complete response and those who did not were performed using the Mann–Whitney U test. Categorical variables were compared using the chi-square test or Fisher’s exact test, as appropriate. All tests were two-sided, and a *p*-value < 0.05 was considered statistically significant.

The primary endpoint was pathological complete response, defined as ypT0N0. An extended definition allowing residual in situ disease in the breast, ypT0/isN0, was evaluated as a secondary endpoint. The primary analyses were based on the ypT0N0 definition.

Inflammatory markers and blood cell counts were analyzed at baseline, before the second treatment cycle, and at the pre-transition assessment. For patients with measurements available at both baseline and the pre-transition assessment, exploratory changes in PIV were calculated as the absolute difference: ΔPIV = PIV pre-transition–PIV baseline.

Percentage change and the natural logarithm of the ratio between pre-transition and baseline PIV were additionally evaluated as sensitivity measures. Corresponding changes between baseline and the assessment before the second cycle were also examined. Between-group comparisons of biomarker changes were performed using the Mann–Whitney U test. Because multiple change metrics and biomarkers were assessed, Benjamini–Hochberg correction was applied to the exploratory between-group change analyses.

The standalone discriminative ability of inflammatory markers was assessed using receiver operating characteristic (ROC) curves and the area under the receiver operating characteristic curve (AUC). Multivariable logistic regression models were used to estimate the probability of pCR. The prespecified clinical reference model included age, Ki-67 index, clinical T stage, and histological grade. Age was modeled per 10-year increase, Ki-67 per 10-percentage-point increase, clinical T stage as T3–T4 versus T1–T2, and grade as G3 versus G2.

Biomarker-extended models were created by separately adding baseline platelet count, baseline PIV, pre-transition PIV, or absolute ΔPIV to the clinical reference model. The clinical reference model included four predictor parameters and 24 pCR events in the complete-case sample, corresponding to approximately 6.0 events per parameter; biomarker-extended models generally included five predictor parameters and 23 pCR events, corresponding to approximately 4.6 events per parameter. For interpretability, platelet count was expressed per 50 × 10^3^/µL increase, whereas PIV and ΔPIV were expressed per 100-unit increase. Odds ratios with 95% confidence intervals (CI) were reported.

To ensure valid incremental comparisons, the clinical reference model was refitted separately on the same complete-case sample used for each biomarker-extended model. Incremental model value was assessed using the likelihood-ratio test and the change in AUC. A paired nonparametric bootstrap procedure was used to estimate 95% confidence intervals for differences in AUC.

Model performance was assessed using apparent AUC with 95% confidence intervals, the Brier score, calibration intercept, and calibration slope. Internal validation was performed using 2000 bootstrap resamples. Optimism was estimated as the mean difference between model performance in each bootstrap sample and performance of the bootstrap-fitted model in the original dataset. Optimism-corrected AUC, Brier score, calibration intercept, and calibration slope were subsequently calculated.

Missing laboratory and clinicopathological data were not imputed. Analyses involving biomarkers were performed using complete cases for the variables included in the respective analysis. The number of patients included in each analysis was reported. Because the availability of the pre-transition assessment depended partly on the treatment sequence, sensitivity analyses were performed in the predominant anthracycline–taxane-based treatment subgroup. Additional exploratory models adjusted for treatment regimen and clinical nodal status were used to assess the robustness of the main findings but were not considered primary models. Initial descriptive and comparative analyses were performed using Statistica v13.3 (TIBCO Software Inc.), whereas the multivariable modeling, paired bootstrap comparisons of AUC, calibration assessment, and bootstrap internal validation were conducted in Python 3.13.

All multivariable analyses were considered exploratory because of the limited sample size and number of pCR events. No model was considered ready for clinical application without external validation.

### 2.5. Patient Characteristics

**Figure 1:**
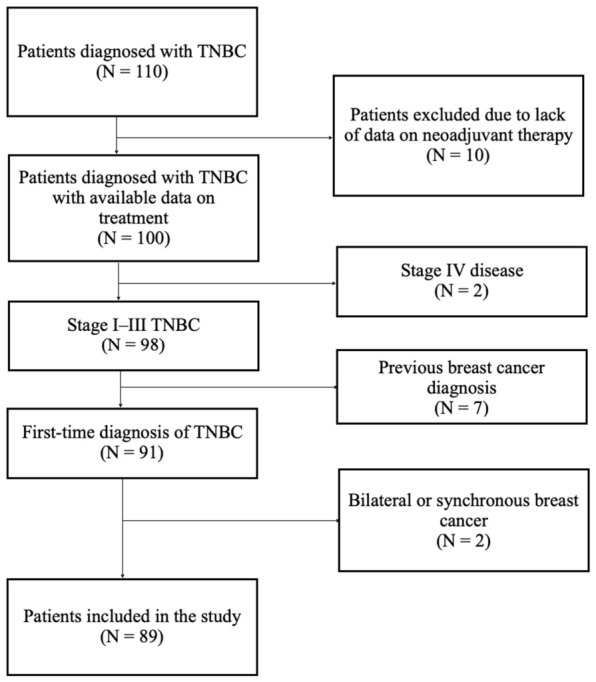
Patient selection flowchart.

We identified 110 TNBC patients who received neoadjuvant treatment. Ten patients were excluded due to missing data on neoadjuvant therapy (treatment received in another hospital); another two patients were diagnosed with stage IV disease (metastases to the lung [N = 1] and liver [N = 1]); seven patients were excluded because they had a previous diagnosis of breast cancer; and two women were diagnosed with bilateral or synchronous breast cancer. Eighty-nine patients were finally included (Figure 1), with a median age of 53.95 years (range 30.54–82.30), a median weight of 67 kg (43–130), a median height of 164 cm (150–179), and a median BMI of 25.49 (17.67–49.54). Arterial hypertension was present in 32.22% of patients, obesity in 20.0%, diabetes mellitus type 2 in 6.67%, and a smoking history in 8.98%.

The predominant neoadjuvant treatment regimen was AC (doxorubicin + cyclophosphamide) followed by a taxane, which was administered to 70 patients (78.65%). Immune checkpoint inhibitor-containing regimens were used in 8 patients (8.99%). Other treatment protocols, including taxane monotherapy, taxane-based combinations, and anthracycline-based variations, were less frequent, each accounting for ≤3.37% of cases (Table 1).

Before neoadjuvant treatment, most tumors were classified as clinical T2 (55.06%), with T1 (5.62%), T3 (22.47%), and T4 (16.85%) being less frequent. Nodal involvement was classified as N1 in 48.31%, N0 in 33.71%, N2 in 16.85%, and N3 in 1.12% of patients. The predominant clinical stage before systemic therapy was IIA–IIB (60.68%), followed by IIIA–IIIC (38.19%), while stage IA accounted for 1.12% of cases. The median Ki-67 index before treatment was 65% (range, 8–99%). Histologic grade G2 was observed in 49.44% of tumors and grade G3 in 48.31%, with 2.25% missing data (Table 2).

After neoadjuvant treatment and surgery, stage 0 was documented in 28.00% of patients, stage IIA in 24.64%, and stage IA–IB combined in 16.80%; advanced stages (IIB–IIIB) represented 22.40% of patients. The median Ki-67 index after treatment was 55% (range, 2–90%). Post-treatment grading showed G2 in 19.10%, G3 in 22.47%, and GX in 25.84%, with 32.58% missing data.

A pathological complete response (pCR), defined as ypT0N0, was achieved in 28.09% of patients, while the extended definition allowing residual in situ disease in the breast (ypT0/isN0) was met by 30.33% of patients. In the overall disease course (disease stage), 74.16% of patients demonstrated tumor regression, 17.98% remained stable, and 7.86% exhibited progression. At the primary tumor level (T status), regression was observed in 76.40%, stability in 21.35%, and progression in 2.25%; regarding nodal status (N status), regression occurred in 35.96%, stability in 49.44%, and progression in 14.61%.

## 3. Results

**Figure 2:**
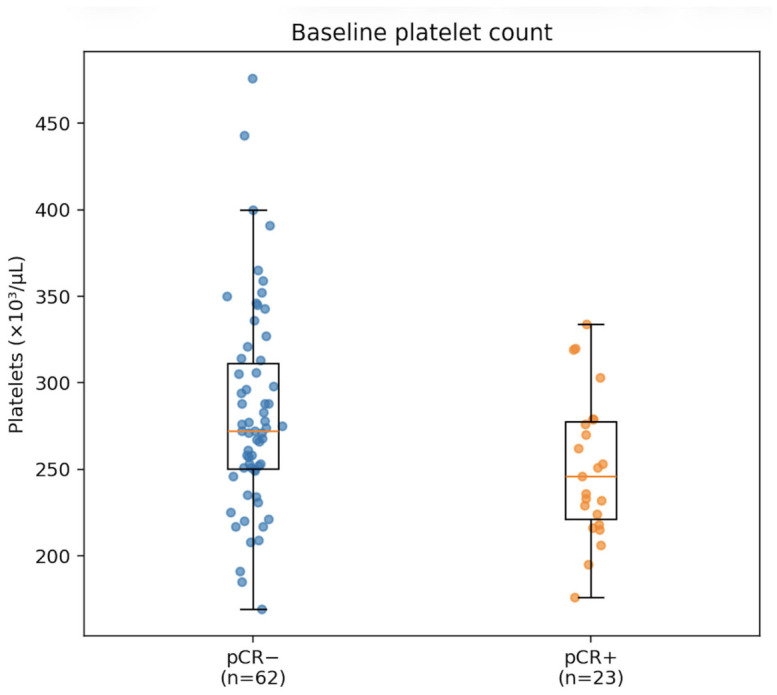
Baseline platelet count according to pathological complete response status. Individual observations are shown together with boxplots. Platelet counts are expressed as ×10^3^/µL. Group sizes are displayed below the *x*-axis. Blue and orange points represent individual observations in the pCR− and pCR+ groups, respectively. The horizontal line inside each box indicates the median.

Baseline laboratory analyses demonstrated that most hematologic parameters measured before the first neoadjuvant treatment cycle did not differ significantly between patients who achieved pCR and those who did not (Table 3). Median neutrophil, lymphocyte, and monocyte counts were comparable between the groups. However, platelet count before the first neoadjuvant treatment cycle was significantly lower in patients achieving pCR than in non-responders (median 246 × 10^3^/µL, range 176–334 vs. 272 × 10^3^/µL, range 169–476; *p* = 0.021). This relationship is presented in the corresponding box-and-whisker plot (Figure 2), where the pCR-positive group showed a lower median platelet count. No significant differences in baseline PIV, NLR, or PLR were observed between patients who achieved pCR and those who did not.

At the second assessment, performed before cycle II, no statistically significant differences were found between responders and non-responders for any of the examined parameters, including platelet, neutrophil, lymphocyte, and monocyte counts, as well as PIV, NLR, and PLR.

**Figure 3:**
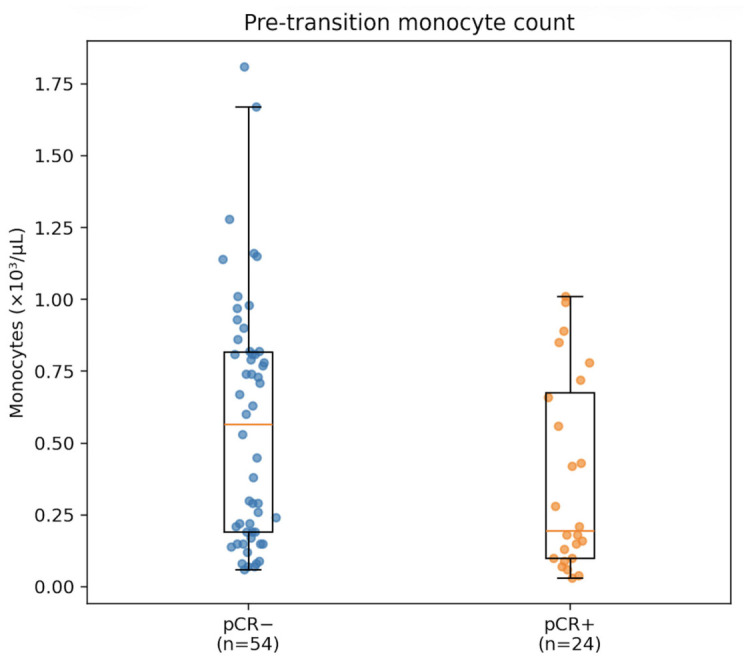
Pre-transition monocyte count according to pathological complete response status. Individual observations are shown together with boxplots. Monocyte counts are expressed as ×10^3^/µL. Group sizes are displayed below the *x*-axis. Blue and orange points represent individual observations in the pCR− and pCR+ groups, respectively. The horizontal line inside each box indicates the median.

**Figure 4:**
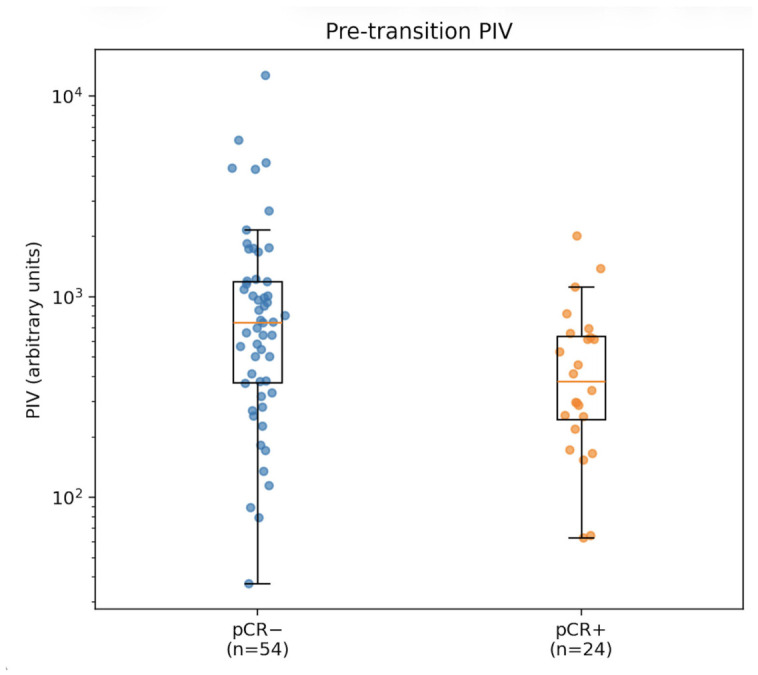
Pre-transition Pan-Immune-Inflammation Value according to pathological complete response status. Individual observations are shown together with boxplots on a logarithmic *y*-axis because of the right-skewed distribution. Group sizes are displayed below the *x*-axis. Blue and orange points represent individual observations in the pCR− and pCR+ groups, respectively. The horizontal line inside each box indicates the median.

At the pre-transition assessment, two parameters showed significant associations with pCR (Table 4). Patients with and without an available pre-transition assessment differed in age and treatment-regimen distribution, indicating that pre-transition missingness was not completely random (Supplementary Table S1). Patients achieving pCR had lower monocyte counts than non-responders (median 0.20 × 10^3^/µL, range 0.03–1.01 vs. 0.57 × 10^3^/µL, range 0.06–1.81; *p* = 0.048). Similarly, PIVs were lower in patients achieving pCR than in non-responders (median 378.9, range 62.8–2019.3 vs. 746.0, range 37.1–12,733.0; *p* = 0.011). These findings are illustrated in Figure 3 and Figure 4. The box-and-whisker plots show lower median values for both parameters in the pCR-positive group, with several high-value observations among non-responders.

Taken together, these results suggest that standard ratios such as NLR and PLR were not significantly associated with pCR, whereas selected components of systemic inflammatory activity—particularly monocyte count and the composite PIV—were associated with treatment response when assessed at the pre-transition time point.

In paired analyses, changes in PIV between baseline and the assessment before cycle II were not associated with pCR. In contrast, the absolute increase in PIV between baseline and the pre-transition assessment was smaller in patients who achieved pCR than in non-responders (median ΔPIV 49.2 vs. 251.4; *p* = 0.043). However, the corresponding percentage change and natural-log ratio did not reach conventional statistical significance (both *p* = 0.059). Furthermore, the association for absolute ΔPIV did not remain significant after Benjamini–Hochberg correction across the exploratory biomarker-change analyses. Therefore, the findings regarding PIV change were considered exploratory. Complete results of the exploratory biomarker-change analyses, including absolute and percentage changes, log-ratios, and Benjamini–Hochberg-adjusted q values, are presented in Supplementary Table S2.

Baseline inflammatory indices demonstrated poor standalone discriminative performance for predicting pCR. The AUC values were close to 0.5 for PIV, NLR, and PLR, indicating limited standalone predictive value.

In the complete clinical model sample of 86 patients, the model incorporating age, Ki-67 index, clinical T stage, and tumor grade achieved an apparent AUC of 0.763 (95% CI, 0.643–0.870). Lower clinical T stage and higher tumor grade were associated with a greater probability of pCR. Following bootstrap internal validation, the optimism-corrected AUC was 0.716, and the corrected calibration slope was 0.779, indicating a degree of model overfitting (Supplementary Table S3).

To evaluate the incremental value of inflammatory markers, each biomarker-extended model was compared with the clinical model refitted on the same complete-case sample (Table 5). Regression coefficients, odds ratios, 95% confidence intervals, and *p*-values for all variables included in the multivariable models are reported in Supplementary Table S4. Among 83 patients with available baseline laboratory data and complete clinical-model variables, the clinical reference model achieved an AUC of 0.751. Addition of baseline PIV produced only a minimal change in discrimination (AUC 0.756; ΔAUC 0.005; bootstrap 95% CI for ΔAUC, −0.015 to 0.068; likelihood-ratio *p* = 0.334). The optimism-corrected AUC of the baseline PIV model was 0.687, indicating no incremental predictive value of baseline PIV.

In the same 83-patient sample, the addition of baseline platelet count increased the apparent AUC from 0.751 to 0.807 (ΔAUC 0.056). The likelihood-ratio test was significant (*p* = 0.023); however, the bootstrap 95% confidence interval for ΔAUC included zero (−0.003 to 0.128). The optimism-corrected AUC of the platelet-extended model was 0.752. These results indicate possible, but uncertain, incremental value of baseline platelet count.

Among 75 patients with complete clinical variables and available pre-transition PIV, the clinical reference model achieved an apparent AUC of 0.735. Addition of pre-transition PIV increased the AUC to 0.798 (ΔAUC 0.063; bootstrap 95% CI, 0.005–0.142; likelihood-ratio *p* = 0.005). The improvement was also retained after optimism correction, with an increase in corrected AUC of approximately 0.063.

**Figure 5:**
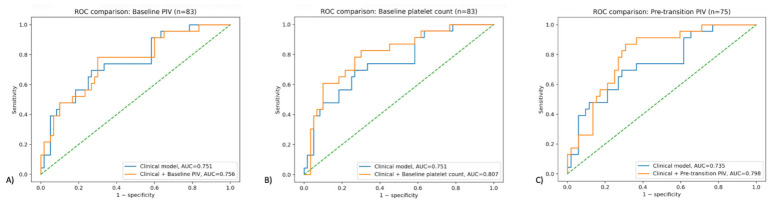
Receiver operating characteristic curves comparing the clinical reference model with biomarker-extended models for predicting pathological complete response. (**A**) Clinical model versus the clinical model plus baseline Pan-Immune-Inflammation Value in the same complete-case sample (n = 83). (**B**) Clinical model versus the clinical model plus baseline platelet count in the same complete-case sample (n = 83). (**C**) Clinical model versus the clinical model plus pre-transition Pan-Immune-Inflammation Value in the same complete-case sample (n = 75). The curves and AUC values represent apparent model performance. Optimism-corrected performance estimates obtained by bootstrap internal validation are reported in Table 5 and Supplementary Table S3. AUC, area under the receiver operating characteristic curve; PIV, Pan-Immune-Inflammation Value; pCR, pathological complete response. The green dashed diagonal line represents the line of no discrimination, corresponding to an AUC of 0.5.

An exploratory model incorporating absolute ΔPIV increased the apparent AUC from 0.735 to 0.807 in the same 75-patient sample. Although the likelihood-ratio test was significant (*p* = 0.003), the bootstrap confidence interval for ΔAUC marginally included zero (−0.001 to 0.140). Because the percentage change and log-ratio analyses were not statistically significant, the ΔPIV model was considered exploratory. The corresponding same-sample ROC comparisons are presented in Figure 5.

Sensitivity analyses restricted to patients receiving an anthracycline–taxane-based regimen showed that the association of pre-transition PIV with pCR remained detectable, whereas the association of baseline platelet count was attenuated. Exploratory adjustment for treatment regimen did not materially alter the direction of the main findings. The distribution of treatment regimens, pCR rates, and availability of the pre-transition assessment according to regimen are presented in Supplementary Table S5.

Overall, baseline PIV did not improve the clinical model. Baseline platelet count showed possible incremental value, although the uncertainty around the change in AUC included no improvement. Pre-transition PIV demonstrated the most consistent incremental association with pCR; nevertheless, all models showed evidence of optimism and should be regarded as exploratory.

## 4. Discussion

Our retrospective study evaluated the role of novel inflammatory markers (PIV, NLR, and PLR), selected complete blood count parameters, and conventional clinicopathological factors in predicting pCR in non-metastatic TNBC patients receiving neoadjuvant treatment. In total, 89 women were included in the analysis.

Our findings indicate that baseline inflammatory biomarkers have limited standalone predictive value in TNBC. Baseline PIV, NLR, and PLR did not differ significantly between patients who achieved pCR and those who did not, and their standalone discriminative performance was poor, with AUC values close to 0.5. Baseline platelet count was the only pretreatment blood-derived parameter that was significantly lower in patients achieving pCR (*p* = 0.021). In contrast, parameters reassessed during treatment, particularly prior to subsequent neoadjuvant therapy (pre-transition), showed significant associations with treatment response. Specifically, lower monocyte counts (*p* = 0.0475) and PIVs (*p* = 0.0111) were observed in patients who achieved pCR.

These findings suggest that the clinical relevance of systemic inflammatory markers may depend not only on their baseline values but also on the timing of assessment during neoadjuvant therapy. Although none of these inflammatory indices alone demonstrated meaningful discrimination for pCR, the conventional clinical model incorporating Ki-67 index, age, clinical T stage, and tumor grade achieved an AUC of 0.76. Lower clinical T stage (T1–T2) and higher tumor grade were associated with a higher likelihood of pCR. The addition of baseline platelet count or pre-transition PIV was associated with a modest increase in model performance, with AUC of 0.81 and 0.80, respectively. These results suggest a potential complementary role of selected blood-based inflammatory parameters, although the exploratory nature of these models and the lack of external validation should be emphasized.

Several studies have explored the relationship between NLR, PLR, and pCR in breast cancer, suggesting that lower pre-treatment levels are associated with increased rates of pCR and improved survival outcomes [25,29,30,31,32]. In a meta-analysis published by Cullinane et al. that included 1586 breast cancer patients who received standardized neoadjuvant treatment, lower NLR was associated with higher pCR rates (odds ratio [OR] 1.83; 95% CI, 1.15–2.91; *p* = 0.0003). Moreover, patients with lower NLR levels had higher 5-year disease-free survival (DFS) (hazard ratio [HR] 1.38; 95% CI, 0.82–2.31); however, statistical significance was not achieved [33]. In contrast, no significant associations between baseline NLR or PLR and pCR were observed in our study. These disparities may be attributed to the biological uniqueness of TNBC and its immunogenicity. Our cohort was limited to TNBC, while the cited studies included populations of heterogeneous breast cancer subtypes (including luminal and HER2-positive subtypes). Due to the relatively small sample size (n = 89), our study may lack the statistical power to detect subtle effects of baseline biomarkers found in larger meta-analyses. However, dynamic assessment of these biomarkers may offer a more nuanced perspective. A clinical model predicting pCR in stage II/III TNBC patients was proposed by Chung et al., this model incorporated presence of an echogenic halo (assessed by breast ultrasound), tumor height-to-width ratio, as well as baseline NLR, baseline PLR and its change during treatment (percentage change in PLR measured before and after the first NAC cycle). The model demonstrated good discriminative performance, with an AUC of 0.877 [34]. These findings suggest that monitoring dynamic changes in biomarkers, rather than single time point measurements, may provide additional predictive value.

In recent years, the predictive and prognostic role of PIV has been increasingly explored. Şahin and colleagues, in a study of 743 breast cancer patients, demonstrated that low pretreatment PIV was an independent predictor of pCR (OR 3.32; 95% CI, 1.53–7.21; *p* = 0.002), and was associated with improved DFS (HR 0.69; 95% CI, 0.49–0.97; *p* = 0.034) and OS (HR 0.61; 95% CI, 0.39–0.95; *p* = 0.028) [35]. Similarly, in a study comprising 1312 patients with stage I–III breast cancer, PIV was identified as an independent predictor of improved OS (HR 1.720; 95% CI, 1.083–2.730; *p* = 0.021). The authors also proposed a prognostic model predicting the 1-, 3-, and 5-year OS, and demonstrated its good discriminative ability. It encompassed variables such as PIV, T stage, N stage, histological breast cancer type, PR status, and Ki-67 [36]. Guclu and colleagues investigated the capability of PIV to predict pCR in a study including 137 patients diagnosed with TNBC, and treated with anthracycline–taxane–based neoadjuvant chemotherapy, with or without carboplatin. Patients with high Ki-67 and low pretreatment PIV (the high Ki-67/low PIV subgroup) demonstrated the highest pCR rates (84%; 31/37), and this subgroup was identified as an independent predictor of pCR (OR 1.88; 95% CI, 1.34–2.97; *p* < 0.001). Moreover, a nomogram including tumor size, clinical N status, chemotherapy regimen and Ki-67/PIV phenotype showed strong discriminatory performance with an AUC of 0.86. However, the main contribution of PIV was the improvement of subgroup-based (rather than global) risk stratification. These findings support the integration of PIV in clinical models predicting pCR after neoadjuvant chemotherapy [37]. In contrast to these findings, we did not observe a significant association between baseline PIV and pCR. The discrepancy may be explained by differences in study populations, treatment regimens, and methodology, but also by the timing of biomarker assessment. While previous studies relied on pretreatment values, our results suggest that lower PIVs measured at the pre-transition assessment were associated with pCR, supporting the concept that dynamic changes in systemic inflammatory markers may provide clinically relevant information. Furthermore, integrating pretreatment platelet count and PIV levels before the next phase of treatment into our conventional clinical model improved its accuracy, with AUCs of 0.81 and 0.80, respectively.

Recently, PIV, NLR, and PLR have emerged as promising and easily accessible markers in various fields of oncology. These ratios were associated with poor prognosis in gastrointestinal tumors, head and neck cancers, as well as advanced lung cancer, supporting their clinical usefulness [38,39,40,41]. Pathological complete response (pCR) was proposed as a surrogate marker of long-term outcomes following neoadjuvant treatment in breast cancer, particularly in TNBC [12,42,43]. In a large meta-analysis comprising 27,895 patients, pCR was associated with improved event-free survival (EFS), especially among patients diagnosed with TNBC [12]. Similarly, a meta-analysis published by Antonini et al. including 12,115 breast cancer patients demonstrated that pCR was associated with longer overall survival (HR 1.30; 95% CI, 1.28–1.33; *p* < 0.00001) and disease-free survival (HR 1.29; 95% CI, 1.24–1.32; *p* < 0.00001). Interestingly, TNBC patients who had pCR after NAC had a 51% increase in OS and a 51% higher DFS compared with those without pCR [42]. In contrast, in a meta-analysis published by Berruti et al. comprising 14,641 breast cancer patients, only weak associations between pCR and long-term outcomes were demonstrated. Therefore, pCR was not recommended as a surrogate endpoint in unselected populations, likely due to the inclusion of heterogeneous breast cancer subtypes and treatment protocols [44]. These findings suggest that pCR remains a clinically meaningful endpoint. However, its validity as a surrogate marker may depend on population homogeneity and tumor subtype, with the strongest prognostic value observed in TNBC.

As described above, achieving pCR after NACT has several long-term benefits. However, underlying tumor biology and mechanisms of chemoresistance influence the efficacy of systemic treatment. Recent preclinical and translational research has explored complex molecular, metabolic, and epigenetic mechanisms through which TNBC cells evade cytotoxic drugs and alter immune responses within the tumor microenvironment. For instance, resistance to taxane-based regimens was linked to epigenetic alterations. Specifically, elevated expression of protein arginine N-methyltransferase 1 (PRMT1), an enzyme involved in regulating posttranslational modifications and influencing oncogenic pathways, was shown to promote the proliferation and migration of TNBC. It was also demonstrated that PRMT1 overexpression contributes to docetaxel resistance, as well as tumor-induced immune suppression via PARP1 methylation [45]. Another mechanism was explored by Dai and Zhang et al., who described the role of circular polo-like kinase 1 (circPLK1), a type of circular RNAs (circRNAs), in resistance to anthracycline-based neoadjuvant chemotherapy in TNBC. The study demonstrated that upregulation of circPLK1 significantly contributes to chemoresistance by regulating the miR-940/ETS1 axis, which was strongly correlated with poor response to NACT and reduced survival among TNBC patients [46]. Furthermore, a study by Duan and Li et al. showed that the interferon regulatory factor 5 (IRF5) gene, highly expressed in TNBC, regulates tryptophan metabolism and promotes TNBC proliferation. The study suggests that targeting the IRF5/solute carrier family 7 member 5 (SLC7A5)/indoleamine 2,3-dioxygenase 1 (IDO1) pathway represents a promising avenue to inhibit tumor progression and potentially improve treatment outcomes [47]. These studies emphasize the importance of further research to better understand the mechanisms by which TNBC develops resistance to chemotherapy and leads to poor survival outcomes.

Several limitations of our study should be acknowledged. Firstly, the findings have limited generalizability due to the single-center and retrospective design, combined with a relatively small sample size. Secondly, although AC (doxorubicin + cyclophosphamide) followed by a taxane constituted the predominant regimen, the inclusion of patients who received immunotherapy, as well as the heterogeneity of chemotherapy protocols may have biased study results. Thirdly, pCR was used as a surrogate endpoint, and long-term outcomes, such as recurrence rates or overall survival, were not assessed. Although changes in PIV were explored using absolute, percentage, and logarithmic measures, the findings were definition-dependent and did not remain significant after correction for multiple comparisons. These analyses should therefore be regarded as exploratory. Finally, the proposed model lacks external validation, which limits interpretation and clinical applicability of our findings.

To the best of our knowledge, no studies have evaluated the integration of pretreatment platelet counts and PIV measured prior to the subsequent treatment (pre-transition) phase into multivariable clinical models predicting response to neoadjuvant treatment in TNBC. Our study results support the concept that assessment of immunological markers at multiple time points may represent a promising avenue in predicting tumor response, thereby improving patient care. However, our model requires further external validation before potential clinical application. Prospective and multicenter studies comprising larger and homogeneous samples are needed to establish the utility of our findings. Moreover, future research should focus on assessing novel therapies in relation to pCR, as well as integrating well-established indicators with emerging biomarkers, such as tumor-infiltrating lymphocytes (TILs), circulating tumor DNA (ctDNA), and genomic signatures and alterations.

## 5. Conclusions

When assessed at baseline, inflammatory markers such as PIV, NLR, and PLR demonstrate limited standalone value in predicting pCR in non-metastatic triple-negative breast cancer. However, combining selected parameters, particularly PIV and platelet counts, with conventional clinical models may improve their predictive accuracy, underscoring the importance of dynamic assessment. Further prospective studies with external validation are needed to confirm their clinical utility and to explore the role of these biomarkers in predicting sensitivity to neoadjuvant systemic treatment.

## Figures and Tables

**Table 1:** Neoadjuvant treatment protocols.

Neoadjuvant Treatment Protocol	N (89)	%
AC (doxorubicin + cyclophosphamide) → taxane	70	78.65
AC (doxorubicin + cyclophosphamide) → taxane + AC	1	1.12
AC (doxorubicin + cyclophosphamide) → taxane + carboplatin	3	3.37
AC (doxorubicin + cyclophosphamide)	1	1.12
TC (docetaxel + cyclophosphamide)	1	1.12
Taxane alone	3	3.37
Taxane + carboplatin	1	1.12
Taxane + doxorubicin	1	1.12
Immune checkpoint inhibitor + taxane + carboplatin → immune checkpoint inhibitor + AC (doxorubicin + cyclophosphamide)	6	6.74
Immune checkpoint inhibitor + taxane + carboplatin	1	1.12
Immune checkpoint inhibitor + AC (doxorubicin + cyclophosphamide) + taxane	1	1.12

**Table 2:** Patients characteristics before neoadjuvant treatment.

Patient Characteristics	N (89)	%
Clinical T stage		
T1	5	5.62
T2	49	55.06
T3	20	22.47
T4	15	16.85
Clinical node status		
N0	30	33.71
N1	43	48.31
N2	15	16.85
N3	1	1.12
Clinical stage before neoadjuvant treatment		
IA	1	1.12
IIA	24	26.97
IIB	30	33.71
IIIA	18	20.22
IIIB	15	16.85
IIIC	1	1.12
Ki-67 before neoadjuvant treatment		
Median, range	65%	8–99%
Histologic grade before neoadjuvant treatment		
G2	44	49.44
G3	43	48.31
Missing data	2	2.25

**Table 3:** Laboratory parameters according to pCR before cycle I (baseline). Data presented as median (range). All blood cell counts are expressed in 10^3^/µL. PIV, NLR, and PLR values are expressed as arbitrary units. Complete laboratory data before cycle I was available for 85 patients. pCR, pathological Complete Response; PIV, Pan-Immune-Inflammation Value; NLR, Neutrophil-to-Lymphocyte Ratio; PLR, Platelet-to-Lymphocyte Ratio.

Parameter	pCR (+)	pCR (−)	*p*-Value
Neutrophils (10^3^/µL)	4.09 (1.56–10.88)	4.15 (0.81–9.26)	0.968
Lymphocytes (10^3^/µL)	1.80 (0.99–3.17)	1.75 (1.03–3.26)	0.645
Monocytes (10^3^/µL)	0.52 (0.25–1.11)	0.57 (0.25–1.10)	0.667
Platelets (10^3^/µL)	246 (176–334)	272 (169–476)	0.021
PIV	341.4 (74.5–2851.5)	338.5 (131.4–1610.7)	0.479
NLR	2.3 (0.7–11.0)	2.4 (0.5–5.9)	0.839
PLR	153.4 (68.0–281.8)	151.3 (69.2–322.9)	0.523

**Table 4:** Laboratory parameters according to pCR at the pre-transition assessment. Data presented as median (range). All blood cell counts are expressed in 10^3^/µL. PIV, NLR, and PLR values are expressed as arbitrary units. Complete laboratory data at the pre-transition assessment were available for 78 patients. pCR, pathological Complete Response; PIV, Pan-Immune-Inflammation Value; NLR, Neutrophil-to-Lymphocyte Ratio; PLR, Platelet-to-Lymphocyte Ratio.

Parameter	pCR (+)	pCR (−)	*p*-Value
Neutrophils (10^3^/µL)	3.94 (1.08–7.46)	4.66 (1.13–14.67)	0.055
Lymphocytes (10^3^/µL)	0.69 (0.20–2.00)	0.83 (0.42–3.13)	0.059
Monocytes (10^3^/µL)	0.20 (0.03–1.01)	0.57 (0.06–1.81)	0.048
Platelets (10^3^/µL)	354.5 (210–655)	364.0 (198–641)	0.630
PIV	378.9 (62.8–2019.3)	746.0 (37.1–12,733.0)	0.011
NLR	6.5 (0.5–24.5)	5.5 (0.7–20.9)	0.841
PLR	572.3 (138.5–1050)	397.4 (121.3–916.3)	0.094

**Table 5:** Incremental performance of biomarker-extended models compared with the clinical reference model on identical complete-case samples. The clinical reference model included age, Ki-67 index, clinical T stage, and tumor grade. For each biomarker comparison, the clinical model was refitted using the same complete-case sample as the corresponding biomarker-extended model. Apparent AUC values are reported together with the change in AUC, paired bootstrap 95% confidence intervals for ΔAUC, likelihood-ratio test *p* values, and the difference in optimism-corrected AUC. AUC, area under the receiver operating characteristic curve; CI, confidence interval; ΔAUC, change in AUC; PIV, Pan-Immune-Inflammation Value; PT, pre-transition assessment.

Biomarker Model	Number of Patients	Number of pCR	Clinical AUC (Same Sample)	Extended AUC	Apparent ΔAUC	Bootstrap ΔAUC CI Lower	Bootstrap ΔAUC CI Upper	Likelihood-Ratio *p*	Difference in Corrected AUC
Baseline platelet count	83	23	0.751	0.807	0.056	−0.003	0.128	0.023	0.052
Baseline PIV	83	23	0.751	0.756	0.005	−0.015	0.068	0.334	−0.013
Pre-transition PIV	75	23	0.735	0.798	0.063	0.005	0.142	0.005	0.063
Absolute ΔPIV	75	23	0.735	0.807	0.072	−0.001	0.140	0.003	0.069

## Data Availability

The data used and/or analyzed during the current study are available from the corresponding author upon reasonable request.

## Supplementary Materials

The following supporting information can be downloaded at: https://www.mdpi.com/article/10.3390/jcm15145614/s1.
