# Supplementary material for: Dynamic Assessment of Systemic Inflammatory Markers in Predicting Pathological Complete Response After Neoadjuvant Treatment in Triple-Negative Breast Cancer
Source: J Clin Med. 2026 Jul 17;15(14):5614. doi: 10.3390/jcm15145614 (PMC13413334; doi:10.3390/jcm15145614)
Supplement: Supplementary file 1 [file jcm-15-05614-s001.zip › jcm-4391536-supplementary.pdf]

Characteristic	Patients available (n=78)	Patients missing (n=11)	Statistical test	p value	Standardized difference
Age, years	51.8 (43.1–65.3)	70.5 (54.1–73.4)	Mann–Whitney U	0.026	-0.776
Ki-67, %	65.0 (40.0–80.0)	40.0 (35.0–75.0)	Mann–Whitney U	0.425	0.283
pCR (ypT0N0)	24/78 (30.8%)	1/11 (9.1%)	Fisher exact	0.171	0.543
cT3–4	30/78 (38.5%)	5/11 (45.5%)	Fisher exact	0.746	-0.142
cN+	49/78 (62.8%)	10/11 (90.9%)	Fisher exact	0.091	-0.666
Grade 3 (G3)	37/76 (48.7%)	6/11 (54.5%)	Fisher exact	0.757	-0.117
Immunotherapy-containing regimen	7/78 (9.0%)	1/11 (9.1%)	Fisher exact	1.000	-0.004
Carboplatin-containing regimen	13/78 (16.7%)	2/11 (18.2%)	Fisher exact	1.000	-0.040
Anthracycline–taxane-based regimen	71/78 (91.0%)	5/11 (45.5%)	Fisher exact	0.001	0.979
Continuous variables are median (IQR). Standardized differences are provided to describe imbalance; p values should not be interpreted as tests of equivalence.					

Supplementary Table S1. Comparison of patients with available and unavailable pre-transition laboratory assessment.

#

Interval	Metric	Marker	n	Number of pCR (+) patients	Number of pCR (-) patients	pCR (+) median change	pCR (-) median change	Between-group p	AUC	BH q	Paired overall p
T0→T1	Absolute	Neutrophils	83	23	60	-0.650	-0.245	0.515	0.453	0.676	0.091
T0→T1	Absolute	Lymphocytes	83	23	60	-0.190	-0.245	0.316	0.428	0.458	0.000
T0→T1	Absolute	Monocytes	83	23	60	0.130	0.205	0.123	0.390	0.259	0.000
T0→T1	Absolute	Platelets	83	23	60	97.000	66.500	0.357	0.566	0.500	0.000
T0→T1	Absolute	PIV	83	23	60	253.060	230.192	0.733	0.475	0.859	0.000
T0→T1	Absolute	NLR	83	23	60	-0.211	0.000	0.803	0.482	0.888	0.134
T0→T1	Absolute	PLR	83	23	60	103.557	63.662	0.043	0.644	0.191	0.000
T0→T1	Percent	Neutrophils	83	23	60	-13.447	-5.637	0.756	0.478	0.859	0.091
T0→T1	Percent	Lymphocytes	83	23	60	-16.964	-14.140	0.220	0.412	0.343	0.000
T0→T1	Percent	Monocytes	83	23	60	20.930	30.611	0.147	0.396	0.280	0.000
T0→T1	Percent	Platelets	83	23	60	42.045	23.713	0.166	0.599	0.280	0.000

#

#

T0→T1	Percent	PIV	83	23	60	81.200	47.678	0.923	0.493	0.939	0.000
T0→T1	Percent	NLR	83	23	60	-8.744	0.000	0.939	0.494	0.939	0.134
T0→T1	Percent	PLR	83	23	60	65.509	45.083	0.026	0.659	0.191	0.000
T0→T1	Log ratio	Neutrophils	83	23	60	-0.144	-0.058	0.756	0.478	0.859	0.091
T0→T1	Log ratio	Lymphocytes	83	23	60	-0.186	-0.152	0.220	0.412	0.343	0.000
T0→T1	Log ratio	Monocytes	83	23	60	0.190	0.267	0.147	0.396	0.280	0.000
T0→T1	Log ratio	Platelets	83	23	60	0.351	0.213	0.166	0.599	0.280	0.000
T0→T1	Log ratio	PIV	83	23	60	0.594	0.389	0.923	0.493	0.939	0.000
T0→T1	Log ratio	NLR	83	23	60	-0.092	0.000	0.939	0.494	0.939	0.134
T0→T1	Log ratio	PLR	83	23	60	0.504	0.372	0.026	0.659	0.191	0.000
T0→PT	Absolute	Neutrophils	77	23	54	-0.550	0.390	0.050	0.358	0.191	0.536
T0→PT	Absolute	Lymphocytes	77	23	54	-0.920	-0.880	0.280	0.421	0.420	0.000
T0→PT	Absolute	Monocytes	77	23	54	-0.250	0.000	0.055	0.361	0.191	0.046
T0→PT	Absolute	Platelets	77	23	54	102.000	72.000	0.164	0.601	0.280	0.000
T0→PT	Absolute	PIV	77	23	54	49.228	251.392	0.043	0.353	0.191	0.000
T0→PT	Absolute	NLR	77	23	54	2.764	2.915	0.480	0.448	0.650	0.000
T0→PT	Absolute	PLR	77	23	54	453.846	231.877	0.043	0.647	0.191	0.000

#

T0→PT	Percent	Neutrophils	77	23	54	-23.002	10.700	0.115	0.386	0.255	0.536
T0→PT	Percent	Lymphocytes	77	23	54	-59.596	-50.604	0.088	0.376	0.216	0.000
T0→PT	Percent	Monocytes	77	23	54	-62.500	0.587	0.042	0.353	0.191	0.046
T0→PT	Percent	Platelets	77	23	54	42.188	26.463	0.086	0.625	0.216	0.000
T0→PT	Percent	PIV	77	23	54	13.455	78.941	0.059	0.363	0.191	0.000
T0→PT	Percent	NLR	77	23	54	106.702	114.381	0.751	0.477	0.859	0.000
T0→PT	Percent	PLR	77	23	54	257.890	167.039	0.026	0.661	0.191	0.000
T0→PT	Log ratio	Neutrophils	77	23	54	-0.261	0.102	0.115	0.386	0.255	0.536
T0→PT	Log ratio	Lymphocytes	77	23	54	-0.906	-0.707	0.088	0.376	0.216	0.000
T0→PT	Log ratio	Monocytes	77	23	54	-0.981	0.006	0.043	0.353	0.191	0.046
T0→PT	Log ratio	Platelets	77	23	54	0.352	0.235	0.086	0.625	0.216	0.000
T0→PT	Log ratio	PIV	77	23	54	0.126	0.581	0.059	0.363	0.191	0.000
T0→PT	Log ratio	NLR	77	23	54	0.726	0.761	0.751	0.477	0.859	0.000
T0→PT	Log ratio	PLR	77	23	54	1.275	0.982	0.026	0.661	0.191	0.000

Supplementary Table S2. Exploratory analyses of changes in inflammatory markers between treatment time points. T0, baseline assessment; T1, assessment before cycle II; PT, pre-transition assessment; pCR, pathological complete response; AUC, area under the receiver operating characteristic curve; PIV, Pan-Immune-Inflammation Value; NLR, Neutrophil-to-Lymphocyte Ratio; PLR, Platelet-to-Lymphocyte Ratio; BH, Benjamini Hochberg correction.

#

Comparison set	Model	n	pCR (n)	Apparent AUC	AUC 95% CI lower	AUC 95% CI upper	Optimism-corrected AUC	AUC optimism	Apparent Brier	Corrected Brier	Apparent calibration intercept	Corrected calibration intercept	Apparent calibration slope	Corrected calibration slope	Successful bootstrap samples	Failed bootstrap samples
Overall clinical model	Clinical reference	86	24	0.763	0.643	0.870	0.716	0.048	0.164	0.186	0.000	-0.148	1.000	0.779	2000	0
Platelets	Clinical reference	83	23	0.751	0.628	0.864	0.700	0.051	0.167	0.191	0.000	-0.160	1.000	0.767	2000	0
Platelets	Clinical + baseline platelets	83	23	0.807	0.691	0.905	0.752	0.055	0.155	0.183	0.000	-0.166	1.000	0.745	2000	0

#

#

Baseline PIV	Clinical reference	83	23	0.751	0.622	0.863	0.700	0.051	0.167	0.190	0.000	-0.168	1.000	0.768	2000	0
Baseline PIV	Clinical + baseline PIV	83	23	0.756	0.634	0.868	0.687	0.069	0.164	0.193	0.000	-0.228	1.000	0.688	2000	0
Pre-transition PIV	Clinical reference	75	23	0.735	0.603	0.852	0.678	0.057	0.179	0.207	0.000	-0.149	1.000	0.749	2000	0
Pre-transition PIV	Clinical + pre-transition PIV	75	23	0.798	0.691	0.893	0.741	0.057	0.163	0.196	0.000	-0.135	1.000	0.723	2000	0
Delta PIV	Clinical reference	75	23	0.735	0.607	0.849	0.679	0.056	0.179	0.206	0.000	-0.156	1.000	0.742	2000	0
Delta PIV	Clinical + absolute ΔPIV	75	23	0.807	0.693	0.900	0.748	0.059	0.159	0.192	0.000	-0.143	1.000	0.722	2000	0

#

#

Supplementary Table S3. Apparent and optimism-corrected performance of the multivariable prediction models. PIV, Pan-Immune-Inflammation Value; AUC, area under the receiver operating characteristic curve; CI, confidence interval.

Model	Predictor	Beta	OR	95% CI lower	95% CI upper	p value
Clinical reference model	Intercept	-0.306	0.736	0.038	14.146	0.839
Clinical reference model	Age (per 10 years)	-0.287	0.750	0.501	1.124	0.163
Clinical reference model	Ki-67 (per 10 percentage points)	0.106	1.112	0.858	1.440	0.424
Clinical reference model	cT3–4 vs cT1–2	-1.285	0.277	0.085	0.904	0.033
Clinical reference model	G3 vs G2	1.152	3.166	1.023	9.799	0.046
Clinical + baseline platelets	Intercept	4.401	81.535	0.302	21988.243	0.123
Clinical + baseline platelets	Age (per 10 years)	-0.423	0.655	0.412	1.042	0.074
Clinical + baseline platelets	Ki-67 (per 10 percentage points)	0.039	1.039	0.783	1.379	0.789
Clinical + baseline platelets	cT3–4 vs cT1–2	-0.915	0.400	0.116	1.377	0.146
Clinical + baseline platelets	G3 vs G2	1.110	3.034	0.949	9.705	0.061

#

#

Clinical + baseline platelets	Baseline platelets count (per 50 ×10³/μL)	-0.693	0.500	0.259	0.966	0.039
Clinical + baseline PIV	Intercept	-0.468	0.626	0.032	12.089	0.757
Clinical + baseline PIV	Age (per 10 years)	-0.265	0.767	0.509	1.157	0.206
Clinical + baseline PIV	Ki-67 (per 10 percentage points)	0.073	1.075	0.828	1.397	0.586
Clinical + baseline PIV	cT3–4 vs cT1–2	-1.374	0.253	0.072	0.889	0.032
Clinical + baseline PIV	G3 vs G2	1.134	3.109	0.987	9.796	0.053
Clinical + baseline PIV	Baseline PIV (per 100 units)	0.060	1.062	0.936	1.206	0.352
Clinical + pre-transition PIV	Intercept	0.021	1.021	0.030	34.236	0.991
Clinical + pre-transition PIV	Age (per 10 years)	-0.146	0.864	0.538	1.389	0.546
Clinical + pre-transition PIV	Ki-67 (per 10 percentage points)	0.083	1.087	0.813	1.452	0.573
Clinical + pre-transition PIV	cT3–4 vs cT1–2	-0.986	0.373	0.104	1.338	0.130
Clinical + pre-transition PIV	G3 vs G2	1.179	3.252	0.953	11.098	0.060
Clinical + pre-transition PIV	Pre-transition PIV (per 100 units)	-0.135	0.874	0.767	0.995	0.041
Clinical + absolute ΔPIV	Intercept	-0.428	0.652	0.022	19.346	0.805
Clinical + absolute ΔPIV	Age (per 10 years)	-0.131	0.877	0.549	1.403	0.585
Clinical + absolute ΔPIV	Ki-67 (per 10 percentage points)	0.054	1.056	0.788	1.415	0.716
Clinical + absolute ΔPIV	cT3–4 vs cT1–2	-1.345	0.260	0.071	0.958	0.043
Clinical + absolute ΔPIV	G3 vs G2	1.236	3.440	0.979	12.088	0.054

#

Clinical + absolute ΔPIV	Absolute ΔPIV (per 100 units)	-0.117	0.890	0.802	0.987	0.028

Supplementary Table S4. Multivariable logistic regression coefficients for the clinical reference model and biomarker-extended models. PIV, Pan-Immune-Inflammation Value; ΔPIV, change in PIV; OR, odds ratio; CI, confidence interval.

**Grouped regimen**	**n**	**Number of pCR**	**pCR rate**	**Number of available patients**	**Patients' availability**
Anthracycline–taxane based	76	23	0.303	71	0.934
Taxane + immunotherapy	8	2	0.250	7	0.875
Taxane based without anthracycline	4	0	0	0	0
Anthracycline alone	1	0	0	0	0

Detailed regimen	n	Number of pCR	pCR rate	Number of available patients	Patients' availability
Taxane + anthracycline	69	21	0.304	65	0.942
Taxane + carboplatin + immunotherapy	7	2	0.286	6	0.857
Taxane + anthracycline + carboplatin	7	2	0.286	6	0.857
Taxane alone	3	0	0	0	0
Anthracycline alone	1	0	0	0	0
Taxane + carboplatin + immunotherapy + cyclophosphamide	1	0	0	1	1
Taxane + cyclophosphamide	1	0	0	0	0

Supplementary Table S5. Treatment-regimen distribution. pCR rates, and availability of the pre-transition assessment according to neoadjuvant treatment type. pCR, pathological complete response.

#

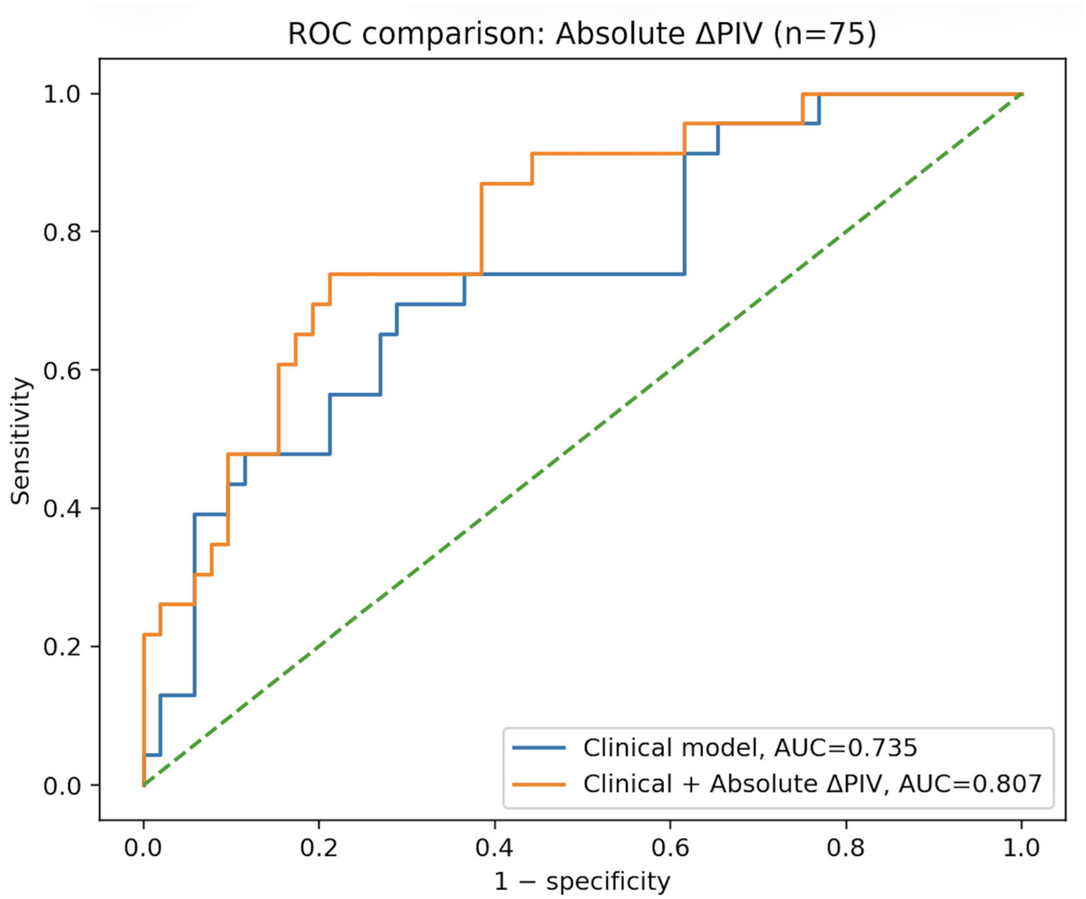

Supplementary Figure S1. Receiver operating characteristic (ROC) curves comparing the clinical reference model with the model additionally incorporating the absolute change in Pan-Immune-Inflammation Value (ΔPIV) from baseline to the pre-transition assessment in the same complete-case sample (n = 75). The curves and areas under the curve (AUC) values represent apparent model performance. PIV, Pan-Immune-Inflammation Value; ΔPIV, change in PIV; AUC, area under the receiver operating characteristic curve.

#

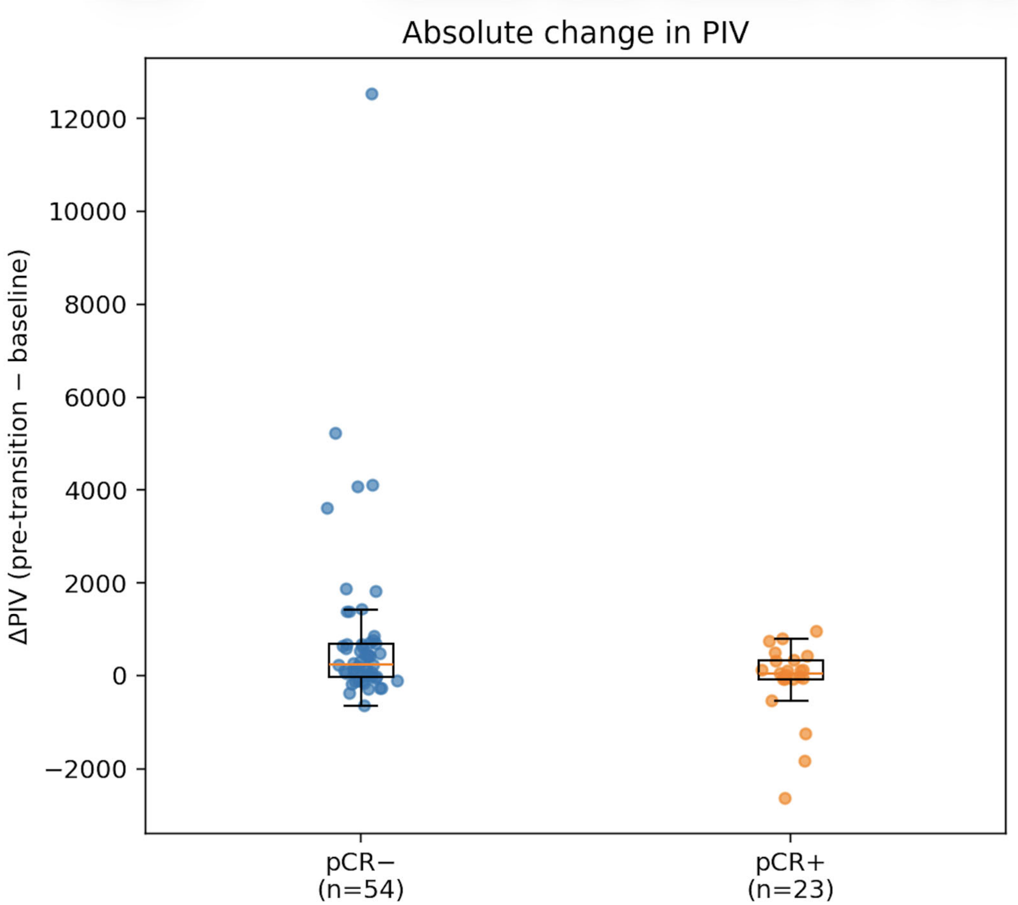

Supplementary Figure S2. Absolute change in Pan-Immune-Inflammation Value from baseline to the pre-transition assessment according to pathological complete response status. Individual observations are shown together with boxplots. Group sizes are displayed below the x-axis. PIV, Pan-Immune-Inflammation Value; ΔPIV, change in PIV; pCR, pathological complete response.
